# Day case laparoscopic cholecystectomy at Kilimanjaro Christian Medical Centre, Tanzania

**DOI:** 10.1007/s00464-020-07914-9

**Published:** 2020-09-01

**Authors:** Imogen Cullen, Fadlo Shaban, Oroog Ali, Matthew Breckons, Kondo Chilonga, Daudi Wapalila, Jamil Suleiman, Mercy Elinisa, Bronwyn Woodburn, Richard Walker, Liam Horgan

**Affiliations:** 1 Newcastle University, Newcastle upon Tyne, United Kingdom; 2 Northumbria Healthcare NHS Foundation Trust, Newcastle upon Tyne, United Kingdom; 3 Institute of Health and Society, Newcastle University, Newcastle upon Tyne, United Kingdom; 4 Kilimanjaro Christian Medical Centre, Kilimanjaro, Tanzania; 5 The Medical School, Newcastle University, Framlington Place, Newcastle upon Tyne, NE2 4HH United Kingdom

**Keywords:** Global surgery, Laparoscopic cholecystectomy, Day case, Low- and middle-income countries, Ambulatory surgery, Tanzania

## Abstract

**Introduction:**

The Lancet Commission on Global Surgery has promoted the case for safe, affordable surgical care in low- and middle-income countries (LMICs). In 2017, Kilimanjaro Christian Medical Centre (KCMC) in Tanzania introduced a day case laparoscopic cholecystectomy (DCLC) service, the first of its kind in Sub-Saharan Africa (SSA). We aimed to evaluate this novel service in terms of safety, feasibility and acceptability by patients and staff.

**Methods:**

This study used mixed methods and was split into two stages. In stage 1, we reviewed records of all laparoscopic cholecystectomies (LCs) comparing day cases and admissions. These patients were followed up with a telephone questionnaire to investigate complication rates and receive service feedback. Stage 2 consisted of semi-structured interviews with staff exploring the challenges KCMC faced in implementing DCLC.

**Results:**

147 laparoscopic cholecystectomies were completed: 109 were planned for DCLC, 82 (75.2%) of which were successful, whilst 27 (24.8%) patients were admitted. No variables significantly predicted unplanned admission, the commonest causes for which were pain and nausea. In the DCLC group there was 1 readmission. 62 patients answered the follow up questionnaire, 60 (97%) of which were satisfied with the service. Stage 2 interviews suggested staff to be motivated for DCLC but revealed poor organisation of the day case pathway.

**Conclusion:**

High rates of DCLC combined with low rates of complications and readmission suggests DCLC is feasible at KCMC. However, staff interviews alluded to administrative problems preventing KCMC from reaching its full DCLC potential. A dedicated day case surgery unit would address most of these problems.

A third of the world’s disease burden comprises surgical conditions. The Lancet Commission on Global Surgery stated in 2015 that total mortality from surgically treatable diseases exceeded the number of deaths caused by malaria, tuberculosis and human immunodeficiency virus combined [1]. In response to this, many low- and middle-income countries (LMICs) have focused their health policies on improving surgical care but there is lack of research evaluating these services [2–6].

In 2017, Kilimanjaro Christian Medical Centre (KCMC) in Tanzania began a day case laparoscopic cholecystectomy (DCLC) service. This service, set up with guidance from a team of surgeons at Northumbria Healthcare NHS Foundation Trust, has benefits for both KCMC and its patients. DCLC improves patient turnover, releases bed spaces, lowers the risk of hospital acquired infection and thromboembolism and allows the patient to return earlier to work [7]. Whilst there is substantial research supporting DCLC available from high income countries (HICs), these data may not be relevant for low resource settings, necessitating the need for research in this area [6]. DCLC has been shown to have some success in North Africa but this is the first service of its kind in Sub-Saharan Africa (SSA) and thus requires thorough evaluation of its feasibility, safety and acceptability by patients [8, 9].

There are often barriers when implementing new procedures in healthcare settings [10]. In HICs such as the UK, day surgery took a long time to become part of common practice due to the traditional mindset promoting inpatient stay after surgery [7]. It was important to assess the barriers in implementing DCLC at KCMC and to understand if the hospital faced mindset issues analogous to the UK. This reflection upon the challenges faced, combined with an evaluation of the service at KCMC, provided evidence for the feasibility of DCLC at KCMC and insight for centres in other LMICs wishing to benefit from DCLC.

## Materials and methods

This was a mixed methods study with quantitative data collected in stage 1 and qualitative data collected in stage 2. In stage 1, theatre logbooks were analysed for total number of patients undergoing laparoscopic cholecystectomy (LC) from October 2017, when DCLC was introduced, to April 2019. Additionally, one patient from 2015, who was conducted as a trial for DCLC feasibility, was included. Patient notes were then retrieved from medical records and examined for evidence of LC done as DCLC along with unplanned admissions, readmissions occurring after discharge and operative details. Telephone numbers were extracted from patient notes. Patients who were both contactable by phone and planned for DCLC received a follow up telephone questionnaire. Post-operative complications such as pain, nausea or infection were assessed using scales of mild, moderate or severe. Patient perceptions of DCLC were sought using closed questions which asked whether patients were satisfied, if they felt safe, if they knew who to contact post-operatively, if they had saved money undergoing DCLC and enquired of their preferred modality of post-operative instructions. Open questions were then used at the end of the call to receive general service feedback so to inquire how the service could be improved. For patients who were planned for DCLC but were admitted, the reason for admission was identified at telephone follow-up to supplement data recorded from the notes.

In stage 2 qualitative data were collected via semi-structured interviews with staff involved with DCLC at KCMC. The staff enrolled included surgeons (who at KCMC also act as ward managers), anaesthetists, ward nurses and scrub nurses. Staff were invited to partake using convenience sampling by visiting the surgical department on different weekdays and weekends. A flexible topic guide was created by IC and DW, a KCMC surgeon, which focused on staff perceptions of barriers and facilitators to DCLCs. Audio recorded interviews were held by IC in English or in Kiswahili using a translator. The transcribed recordings were analysed using the principles of thematic analysis [11].

Ethical clearance was granted locally from KCMC. Informed consent was obtained for telephone calls and staff interviews. Data were stored on an encrypted database.

## Results

One hundred and forty-seven LCs were collected from the logbook: 109 (74.1%) were planned for day case, 82 (75.2%) of which were successfully operated on as day case whilst 27 (24.8%) were admitted. Of the 27 inpatients, 5 (18.5%) were admitted the day before surgery, 13 (48.1%) were admitted after surgery and 9 (33.3%) were admitted before surgery and remained inpatients post-operatively.

All operations included were led by one of three consultants—all of whom had varying levels of laparoscopic experience (Table 1).

**Table 1:** Table ranking consultants at KCMC by no. of laparoscopic operations performed whereby surgeon 1 is the most experienced and surgeon 3 is the least experienced

Consultant	Number of LCs performed since 2015
Surgeon 1	81
Surgeon 2	52
Surgeon 3	8

The most experienced surgeon, Surgeon 1, performed the majority, 50 (61.0%), of the successful DCLC. Surgeon 2 and 3 performed 26 (31.7%) and 2 (2.4%) respectively. A UK consultant performed 1 (1.2%) DCLC and data were missing for 3 (3.7%) operations. Of the 27 inpatient LCs a lower percentage were performed by Surgeon 1, 13 (48.1%). Surgeon 2 and 3 performed 10 (37.0%) and 2 (7.4%) respectively and data were missing for 2 (7.4%) operations.

The unplanned admissions both before and after surgery were counted as separate events. Therefore, the sum of unplanned admissions was calculated by adding these, totalling 36. Five (13.9%) patients were admitted the day before surgery with the reason being that they were first on the list the following day, 2 (5.6%) were unsure why they were admitted a day prior to surgery. Post-surgery, 3 (8.3%) were admitted due to late surgery finish, 5 (13.9%) due to conversion to open surgery, 3 (8.3%) due to unmanageable pain, 3 (8.3%) due to nausea and vomiting, 1 (2.8%) due to hypotension, 1 (2.8%) patient was recorded to be ‘still under the influence of anaesthetic’ and 13 (36.1%) remained unknown.

Successful DCLC and failed DCLC were analysed for medical and demographic factors predicting admission (Table 2).

**Table 2:** Medical and demographic factors in those with successful, and failed, DCLC

Factors	DCLC	LC	Significance	Missing DCLC	Missing LC
Median ASA	1	1	*U* = 0.789, *p* = 0.587	12	1
Mean age	43.6 ± 12.8	44.7 ± 13.7	*T* = 0.36, *p* = 0.716	4	1
Sex M:F	10.7: 1	7.3: 1	*χ*^2^ = 0.271, *p* = 0.602	0	0
Median comorbidities	0	0	*U* = 0.412, *p* = 0.412	0	0
Operation time (min)	77	92	*T* = 1.944, *p* = 0.055	5	2
Mean morphine given (g)	7.6	9.5	*T* = 1.276, *p* = 0.205	5	2
Mean distance lived from KCMC (km)	181	127	*T* = 1.160, *p* = 0.249	8	3

Thirty-seven (59.7%) of the 62 successful DCLC patients followed up with a phone call reported discharge home with pain. Of these, 1 (2.7%) reported severe pain, 19 (51.4%) described moderate pain and 7 (18.9%) described minimal pain. Ten (27.0%) patients could not describe or remember the nature of the pain. Thirteen (21.0%) DCLC patients reported post-operative nausea and vomiting (PONV), of whom, 2 (15.4%) felt they were unable to control this at home.

Seventy-two (87.8%) of DCLC and 25 (92.6%) of LC patients were recorded to receive prophylactic antibiotics. Seven (11.3%) DCLC patients reported surgical site infection (SSI) after surgery, of whom 6 received additional outpatient treatment and 1 was readmitted (the only readmission of the cohort). Three (17.6%) unsuccessful DCLC patients reported SSI post-operatively however, this difference in SSI post-operatively between DCLC and inpatients was not significant (*χ*^2^ = 1.292, *p* = 0.256).

Sixty-one (98%) of the DCLC patients were either “satisfied” or “extremely satisfied”. One patient was not satisfied due to poor counselling and ward conditions. 62 (100%) were able to rest adequately at home and also would recommend to family and friends. Fifty-seven (91.9%) felt safe at home and 57 (91.9%) saved money by having day surgery. Fifty-one (82.3%) knew whom to contact post-operatively. When asked “is there anything you would like to see change in the service” 7 (11.3%) patients flagged the issue of poor patient counselling. Patients were asked if they would have liked to receive clearer post-operative instructions upon discharge. Of the 60 (97%) who answered yes, 29 (48%) preferred a leaflet, 23 (38%) a text message, 5 (9%) an email, 2 (3%) verbal instruction and 1 (2%) a follow up phone call.

Twenty-six qualitative interviews were conducted with: 8 scrub nurses, 7 surgeons, 5 anaesthetists and 6 ward nurses. The 4 major themes identified were: (i) patient factors, (ii) organisation of the day case pathway, (iii) staff mindset and (iv) low resources. Quotations from individuals were identified using a unique code specific to their vocational group, followed by a number:

- WN: ward nurse
- S: surgeon/ward manager
- A: anaesthetist
- SN: scrub nurse

*Patient factors* affecting the feasibility of day case surgery were described by staff. This included patient attitudes and behaviour resulting in hospital admission where fearful patients resisted same day discharge. This was felt by some to be linked to a lack of counselling in advance of the surgery:

WN4 “Patients fail to be taught what does this [day case] mean?”

Other staff highlighted that the distance patients lived from KCMC made same day discharge challenging:

S1 “When we told them that you should go home the same day they are very reluctant to go home because they live quite far-away”

Several factors were identified around the *organisation of the day case pathway* which reduced the overall efficiency and resulted in avoidable hospital admissions. Organisation was an issue for the pre-and post-operative stages as well as the day of surgery. For example, poor planning pre-surgery contributed to many patients receiving their pre-operative anaesthetic review in the corridor often without any blood results. This sometimes resulted in delayed post-operative recovery and unplanned admission:

A4: “She was admitted for the night. It was delayed recovery that occurred… We did not know how the liver was functioning, maybe we would have given less drugs”

On the day of surgery there was no day case specific theatre room, list or ward at KCMC. This meant day case patients were mixed with inpatients and were subject to delays secondary to emergencies or complicated elective operations:

SN3: “There was an idea of constructing some extra (day case) rooms, but it is a big project… it needs a lot of money, so the plan has to wait”

This comment described an acknowledgement of the problem, but a potential solution (the construction of day case rooms) was unfeasible due to resource constraints. Post-operatively discharging patients was difficult as the hospital was not prepared for same day discharges which occurred later in the day than routine inpatient discharge. Where the patient was paying by cash discharge was difficult as the cashiers responsible for processing payment closed at 5 pm:

WN3: “Where the patient is discharged and is paying cash, the cashiers are counting the cash, but those cashiers close at 5 pm”

Whilst all staff reported day surgery as safe and beneficial to patients, some criticism was directed at the *mindset* of medical records staff and new members of staff, suggesting that fixed ideas held regarding discharge impacted on DCLC efficiency:

S5: “These intern doctors feel that once the patient has been operated, they at least should be monitored in the hospital for a day or two before discharged.”

S3: “Take medical records, sometimes they become uncomfortable when patients are being discharged past evening hours… What they believe is that there is a ‘discharging time’ for the patients and that is fixed in their mind. That you have to change.”

*Low resources* were a recurring and cross-cutting theme with data highlighting that financial resource was not available to invest in the issues surrounding *patient factors, organisation of the day case pathway* or *mindset*. Other practical issues relating to *low resources* were reflected by the anaesthetists who described a lack of short acting drugs resulting in the use of longer acting drugs. This could have caused delayed post-operative recovery contributing to unplanned admissions:

A3: “If we have fentanyl, we give it to them but if it is not available we tend to use morphine”

The issue *low resources* was embodied also in the description of the lack of staff. This was highlighted as a logistical factor which could lead to patients being admitted overnight due to no one having time to discharge them:

S5: “The last one is man-power. Ward manager is dying here. Literally dying. Taking care of sixty patients every day, planning the list every day, it's tough”

## Discussion

There are no national guidelines in Tanzania providing targets for the number of LC that should be DCLC. The success rate from KCMC (55.8%) was around double that of the data found in North Africa (22.6%, 28.5%) [8, 9]. Guidelines from HICs such as the UK recommend DCLC rates to be above 75% but average UK DCLC rates in 2017 (55%) matched those seen at KCMC in this study [12, 13]. The cause of this high rate is likely multifactorial. Firstly, KCMC has developed its DCLC service with the support of an established, thorough and sustainable training scheme with Northumbria Healthcare [14]. Secondly, it may be due to the operations being entirely consultant lead, with the most experienced consultant performing the majority of all LC’s. This would contrast to the UK where consultants may supervise but not lead the surgery [15]. It also may be, in part, due to the fact that KCMC do not operate on acute cholecystitis. A UK study showed that adverse outcomes in LC were associated with increased urgency and complexity of surgery [16]. Excluding acute cholecystitis at KCMC meant the LCs performed were less complex and day case more achievable. This contrasts to the UK where NICE guidelines recommend a person presenting with acute cholecystitis receive LC within 7 days [17]. The UK’s 55% is calculated as a mean, so high volume centres who operate on a high number of acute gallbladders may lower the overall mean. The two sites are difficult to compare. Although KCMC’s results may be partly due to not operating in acute cholecystitis, these impressive results suggest that the introduction of this new service has been successful. The rate of uptake is particularly promising considering these figures come only two years after DCLC was first offered. For comparison, in 2008 in the UK, around 10 years after the start of day case LC and before acute gallbladder guidelines were introduced; the average DCLC rate was 16% [18].

At KCMC the top reasons for admission post-surgery reflected complications of anaesthesia; nausea and vomiting, hypotension and patients remaining under the influence of anaesthetic. These data suggested an issue with anaesthetic techniques chosen at KCMC. The mean grams of morphine used in DCLC and LC were 7.6 and 9.5, respectively. Although morphine was not a predictive factor for admission, this was likely due to it being used consistently across LC and DCLC at KCMC. Patients have heterogeneous reactions to morphine dosages, this made statistical significance difficult to achieve in a study sample of this size. Qualitative data from staff suggested that choice of anaesthetic may sometimes be based on availability rather than clinical indication and hence the use of short acting medication such as fentanyl, which has the potential to improve DCLC rates, may not always be feasible. This highlights the unique challenges that face KCMC and other LMICs in implementing DCLC. Training at these centres may need to be adapted to include how best to manage a patient without access to the gold standard medications and importantly, when DCLC may be inappropriate due to lack of resources.

Some readmissions after DCLC are unavoidable, but excessive rates can indicate poor surgical skill or inappropriate discharge. However, rates here are similar to that seen in HIC centres and suggests DCLC at KCMC is safe [19]. The number of cases of severe pain is less in this study (2.7%) than found in a similar study based in a HIC by Kavanagh et al. (23%) suggesting patients at KCMC are being discharged with appropriate analgesia [20]. Despite Kavanagh et al. not using morphine they reported PONV (25%) to be similar to that seen at KCMC (21%). This may be due to an increased severity of PONV at KCMC, resulting in patient admission thus reducing the overall rates of PONV reported in DCLC. A more thorough assessment of PONV may be needed to clarify its burden at KCMC.

SSIs were higher in the DCLC group (11.3%) compared to data available from the UK (0.5%).[21]. This is likely to be due to a multifactorial causality. Nutrition plays a major role in wound healing and Tanzania suffers a double burden of rising obesity rates in a population where undernutrition still prevails [22, 23]. Compromised immune systems also contribute to SSI rates. The HIV prevalence in Tanzania sits at 4.6% whereas the average in the UK is 0.16% [24, 25]. This increased national rate may have influenced the rates of SSI at KCMC. Knowing the exact prevalence of HIV in this study population would have allowed a more accurate analysis. Additionally, KCMC uses reusable laparoscopic equipment. Whilst a systematic review showed infection rates to be similar between disposable and reusable equipment, this was providing the equipment was cleaned properly between use [26]. Our study did not include an audit of cleaning protocol or technique. It is therefore be difficult to conclude if this had contributed to the higher infection rates. It is also difficult to comment on the role of prophylactic antibiotics. Not all patients are recorded as receiving antibiotics which is likely to be due to missing data from the operation note rather than its role as a true negative.

The difference between the SSIs reported in the DCLC and inpatient groups was not significant (*p* = 0.249). This may be due to small sample size or could be due to the lack of a dedicated day case surgery unit. Inpatients were mixed with day cases on one overcrowded ward, placing DCLC patients at higher risk of acquiring an infection from inpatients.

The majority of patients reported a positive experience. The questionnaire used included a larger range of questions than previous work conducted in LMICs on day case surgery and demonstrated patients were not only satisfied but felt safe, could rest adequately at home and had saved money using the service [8, 9]. This is important, as it has been shown in HICs that patient satisfaction is high for day surgery but this was yet to be shown or to be comprehensively explored within an LMIC culture [27]. Patients did however highlight the need for more information. It has previously been of concern that printed instructions may be inappropriate due to low literacy rates in Tanzania, yet the feedback obtained in this study suggests patients would have benefited from a leaflet [28]. The fact that staff described a lack of patient understanding as a potential barrier to day case treatment suggests the need for improvement of patient education and counselling.

The medical and social characteristics of the DCLC patients and inpatients were similar. Although previous studies have reported operation time to be an independent predictor for unplanned admission, in this study operation time was not significant (*p* = 0.055) [29, 30]. The lack of significant predictive factors for admission measured, suggests other aspects may be involved. Stage 2 suggested additional issues perceived to be preventing discharge. For example, the distance that some patients live from the hospital makes travelling home after surgery not logistically possible for everyone. This has been described in other LMICs where patients who travel long distances to attend surgery are excluded from day case eligibility [31, 32]. Reasons why patients were not planned for DCLC in the first instance was not included in this study but may have offered further evidence that distance lived from the hospital hindered access to day case services. Additionally, prevailing views of some groups of staff who were not interviewed in this study, such as new interns and medical records staff, that patients should be admitted to hospital for LC were barriers to discharge. This perhaps highlights the need for additional training or the introduction of policy or guidelines. This would improve the structure of the care pathway that was discussed as a potential reason behind unplanned admission of patients. Clearer protocols may also see improved candidate selection, timely processing of blood results, improvements in discharge policy and better mark DCLC patients separate from inpatients; all issues which were cited by staff as barriers to DCLC.

A major strength of this study is that it offers a coherent analysis of several features of DCLC rather than focusing on surgical outcomes, patient perceptions or staff attitudes alone. The mixed methods design used in this study allowed a thorough and detailed analysis of the setup of DCLC at KCMC from the perspectives of multiple stakeholders. The use of qualitative survey methods provided a richer understanding of the data observed. To the best of our knowledge this is the first study of its kind to be conducted regarding DCLC in SSA. However, retrospective data collection were challenging. Handwritten records were sometimes illegible, so some data were missing. These data were not associated with any confounding variables to the best of the authors knowledge.

All telephone interviews were subject to recall bias and patients may only have been able to remember symptoms if they were severe. There was patient attrition between medical records and telephone follow up, with many patients being unreachable. This may have been due to patient death, which would be more likely in frailer patients or those whose LC had resulted in a major complication, so this study sample may not be reflective of the overall cohort population.

The nature of thematic analysis is that the researcher is embedded in the data and responsible for the formation of key concepts that evolve from the findings making conclusions subjective. Subjectivity resulting in researcher bias could have been reduced by having a second coder involved in the analysis. However, it has been suggested that whether coding is carried out by a lone researcher or by a team holds less significance than the importance of using a systematic process to analyse the data which is made to be transparent.

## Conclusion

This study has shown that at KCMC, DCLC is feasible. The rates of DCLC per total LC show that KCMC has impressive capacity to achieve such a service despite its low resource setting and the low rates of readmission indicated DCLC to be safe. The telephone follow-up revealed that the patients benefitted from DCLC and were accepting of same day discharge.

Contrary to expectations, staff at KCMC were accepting of DCLC. It was found that many of the challenges seen in achieving DCLC at KCMC were underpinned by low resources and poor organisation. The lack of a day case specific ward prevented lowered infection rates, absence of printed protocols resulted in poor patient flow and limited availability of anaesthetic agents with the widespread use of morphine reduced DCLC efficacy. To improve the service at KCMC the day case patient pathway needs to be strengthened. This may be through developing a day case protocol and building a day case unit, which we are pleased to report is underway.

This study provided insight into the challenges LMICs may face in setting up DCLC. The results suggest that DCLC may be feasible in other LMICs and pave the way for successful service development and sustainability in the drive for accessible global surgery.
