# Correction to: Day case laparoscopic cholecystectomy at Kilimanjaro Christian Medical Centre, Tanzania

**DOI:** 10.1007/s00464-021-08324-1

**Published:** 2021-01-21

**Authors:** Imogen Cullen, Fadlo Shaban, Oroog Ali, Matthew Breckons, Kondo Chilonga, Daudi Wapalila, Jamil Suleiman, Mercy Elinisa, Bronwyn Woodburn, Richard Walker, Liam Horgan

**Affiliations:** 1 Newcastle University, Newcastle upon Tyne, United Kingdom; 2 Northumbria Healthcare NHS Foundation Trust, Newcastle upon Tyne, United Kingdom; 3 Institute of Health and Society, Newcastle University, Newcastle upon Tyne, United Kingdom; 4 Kilimanjaro Christian Medical Centre, Kilimanjaro, Tanzania; 5 The Medical School, Newcastle University, Framlington Place, Newcastle upon Tyne, NE2 4HH United Kingdom

## Correction to: Surgical Endoscopy 10.1007/s00464-020-07914-9

This article was updated to correct the spelling of Jamil Suleiman’s name in the author listing and Disclosures.

